# Computing of temporal information in spiking neural networks with ReRAM synapses

**DOI:** 10.1039/c8fd00097b

**Published:** 2018-07-11

**Authors:** W. Wang, G. Pedretti, V. Milo, R. Carboni, A. Calderoni, N. Ramaswamy, A. S. Spinelli, D. Ielmini

**Affiliations:** a Dipartimento di Elettronica, Informazione e Bioingegneria , Politecnico di Milano , Piazza L. da Vinci , 32 – 20133 Milano , Italy . Email: daniele.ielmini@polimi.it; b Micron Technology, Inc. , Boise , ID 83707 , USA

## Abstract

Resistive switching random-access memory (ReRAM) is a two-terminal device based on ion migration to induce resistance switching between a high resistance state (HRS) and a low resistance state (LRS). ReRAM is considered one of the most promising technologies for artificial synapses in brain-inspired neuromorphic computing systems. However, there is still a lack of general understanding about how to develop such a gestalt system to imitate and compete with the brain’s functionality and efficiency. Spiking neural networks (SNNs) are well suited to describe the complex spatiotemporal processing inside the brain, where the energy efficiency of computation mostly relies on the spike carrying information about both space (which neuron fires) and time (when a neuron fires). This work addresses the methodology and implementation of a neuromorphic SNN system to compute the temporal information among neural spikes using ReRAM synapses capable of spike-timing dependent plasticity (STDP). The learning and recognition of spatiotemporal spike sequences are experimentally demonstrated. Our simulation study shows that it is possible to construct a multi-layer spatiotemporal computing network. Spatiotemporal computing also enables learning and detection of the trace of moving objects and mimicking of the hierarchy structure of the biological visual cortex adopting temporal-coding for fast recognition.

## Abstract 2

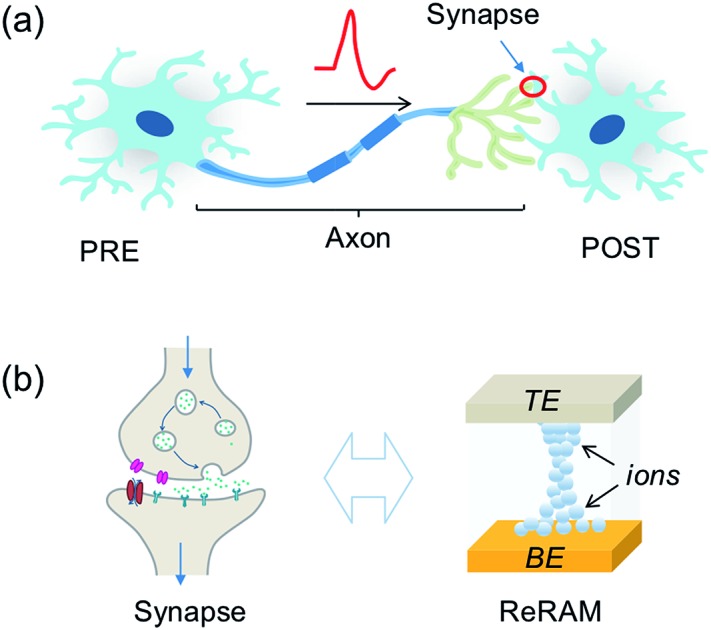
This work addresses the methodology and implementation of a neuromorphic SNN system to compute the temporal information among neural spikes using ReRAM synapses capable of spike-timing dependent plasticity (STDP).

## 1 Introduction

The most relevant advances of artificial intelligence (AI) are currently in the area of deep neural networks (DNNs),^1^ which enable the learning and recognition of images, sounds, and speech. Despite the broad success of DNNs, their supervised training requires a huge amount of computational resources, while their energy consumption is several orders of magnitude higher than the human brain. Since DNNs are implemented in conventional computers, such as the graphic processing unit (GPU) utilizing the complementary metal-oxide-semiconductor (CMOS) technology, the slowing down of Moore’s law may create a critical issue for the future progress of DNNs. Another possible roadblock for conventional computers is the memory bottleneck, arising from the large latency and energy consumption due to the physical separation between memory and computing circuits according to the von Neumann architecture. The memory bottleneck critically affects the implementation of DNNs, which are inherently data hungry.^2^ On the other hand, the brain adopts an in-memory computing approach, where there is no distinction between memory and computing units. Neuromorphic computing takes inspiration from the brain to achieve a higher energy efficiency than software-based DNNs for solving AI tasks.^3^,^4^ Resistive memory devices,^5^,^6^ including resistive random-access memory (ReRAM) and phase change memory (PCM), can be used as artificial synapses with analog plasticity, similar to biological synapses.^7^–^13^

Resistive switching synapses are used in neuromorphic systems according to two approaches. In the first approach, an artificial neural network (ANN) is trained by supervised learning, *e.g.*, the backpropagation (BP) algorithm,^14^–^18^ to construct a hardware accelerator for DNNs.^19^–^21^ The major issue for this approach is the non-linear weight update and the large variability of resistive switching devices.^20^,^22^ On the other hand, brain-inspired spiking neural networks (SNNs) aim at replicating the brain structure and computation in hardware. Learning usually takes place *via* spike-timing dependent plasticity (STDP),^23^–^27^ where synapses can update their weight according to the timing between spikes of the pre-synaptic neuron (PRE) and post-synaptic neuron (POST). This approach provides a more biologically plausible way to implement neuromorphic computing. Spikes convey information which can be coded in their rate, namely a high rate of the spikes represents a high intensity of the external/internal signal,^28^ or by more efficient types of coding,^29^,^30^ namely spatiotemporal coding^31^,^32^ or precise spiking coding.^33^–^35^ Spatiotemporal coding, in particular, contains information about space (which neuron is spiking) and time (when a neuron is spiking in relation to other neurons). The neuron corresponding to stimuli with the highest intensity spikes first, while neurons with lower intensity spike later. Spatiotemporal coding is a sparse coding method with high information capacity, and the neural systems based on such coding method theoretically show much larger solution space than the perceptron-based ANN.^36^,^37^

Here we aim at addressing the methodology and implementation of neuromorphic computing based on spatiotemporal coding, also exploring possible applications of temporal computation using ReRAM synapses. We proposed a hybrid system combining CMOS neurons and ReRAM synapses. STDP is achieved in ReRAM synapses for both long-term potentiation (LTP) and long-term depression (LTD), while CMOS neurons provide the necessary spiking excitation for realizing plasticity. We first experimentally demonstrate the learning and recognition of spatiotemporal-coded spike sequences, enabling the wide application potential, *e.g.*, spell checking and DNA analysis. Multi-layer spatiotemporal computing within ReRAM synaptic arrays is also presented. We show that spatiotemporal computing can enable learning and detection of the trace of a moving object. We then design and simulate a pattern recognition system mimicking the hierarchy structure of the biological visual cortex. The results confirm the ability of the temporal computing of ReRAM synapses and the feasibility of ReRAM synapses for the implementation of a brain-like neuromorphic system with efficient spatiotemporal coding.

## 2 Results and discussion

### 2.1 ReRAM device as an artificial synapse

**Fig. 1:**
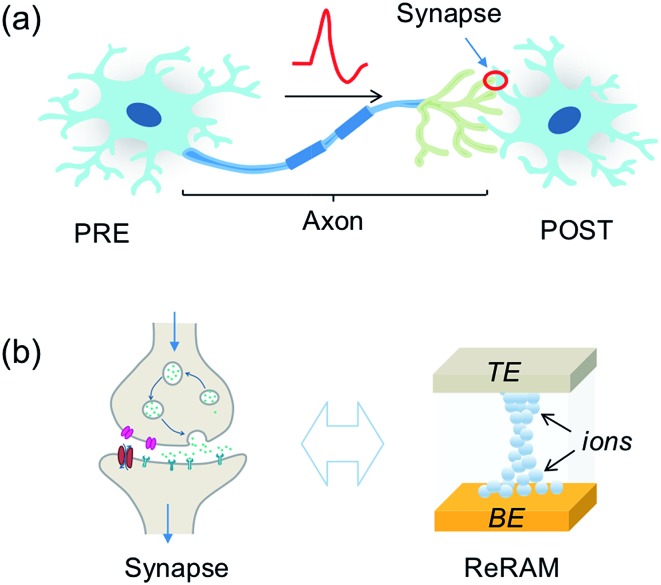
(a) Illustration of a building block of the biological neural system, consisting of a pre-synaptic neuron (PRE), a post-synaptic neuron (POST), and a synapse between the PRE axon and POST dendrite. (b) Comparison between the biological synapse (left) and electronic synapse (right, resistive switching memory, ReRAM).

Despite the complexity of the human brain, which still defies understanding nowadays, the individual building blocks in the brain are relatively well known, as shown in Fig. 1a. Here, a PRE is connected to a POST *via* a synapse between the PRE axon and the POST dendrite. The synapse dictates the amount of signal passing from the PRE to the POST, according to the synaptic weight. The latter can be modified throughout the life of the synapse by plasticity, which is responsible for the learning process of the brain. The signal transmission, weighting and plasticity behavior can be mimicked by the two-terminal, non-volatile, and nanoscale ReRAM device shown in Fig. 1b.

The biological synapse weights the signal from the PRE by releasing a certain amount of neurotransmitters and activating the dendrite membrane with receptors. The synaptic plasticity derives from the regulation of the amount of neurotransmitters and the number and distributions of receptors. On the other hand, the ReRAM synapse can be viewed as a variable conductance which transforms a voltage signal into a current proportional to the synaptic conductance, which thus plays the role of the weight. The ReRAM conductance can be modified by a higher voltage excitation, thus mimicking the plasticity of the biological synapse.^8^,^38^

### 2.2 Computation of the temporal correlation of spikes

**Fig. 2:**
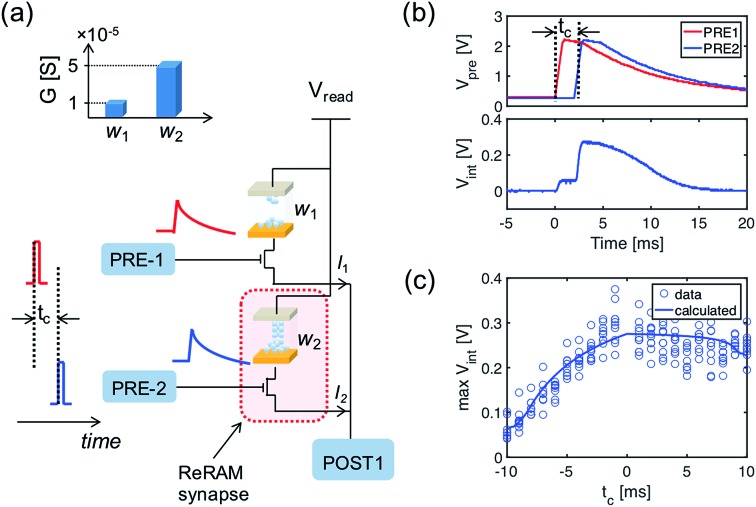
(a) Schematic view of the temporal computation between the two spikes using ReRAM synapses. To enable the interference of the two temporally separated PRE spikes, the PREs convert the spike signals into exponentially decaying voltage signals applied to the gate of the one-transistor/one-ReRAM (1T1R) synapses. Inset: the conductance of the two ReRAM devices. (b) The exponentially decaying signals of the output of the two PREs (upper panel) and the internal potential (*V*_int_) of the POST (lower panel). (c) Maximum *V*_int_ as a function of the temporal correlation (the time difference of the two PRE spikes, *t*_c_).

The computation of the temporal information among spikes is illustrated by the computation of the temporal correlation between the two spikes in Fig. 2. Assuming a simple network of 2 PREs, 2 synapses, and 1 POST (Fig. 2a), the temporal correlation between the PRE spikes can be denoted as their time delay *t*_c_. The CMOS PRE circuits convert the spikes into exponentially decaying pulses, mimicking the shape of the action potential reaching the axon (Fig. 1a). Each exponential pulse *V*_G_ is applied to the gate of a one-transistor/one-ReRAM (1T1R) synapse, and is given by:1
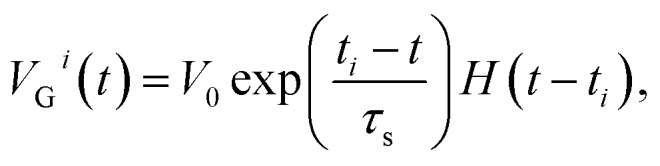
where *t* is time, *t*_*i*_ is the spiking time of the *i*-th PRE, *τ*_s_ is the decay time constant of the exponential pulse, *V*_0_ is a parameter controlling the maximum value of the signal, and *H*(*t*) is the Heaviside function. The voltage applied on the gate terminal excites a synaptic current given by:2
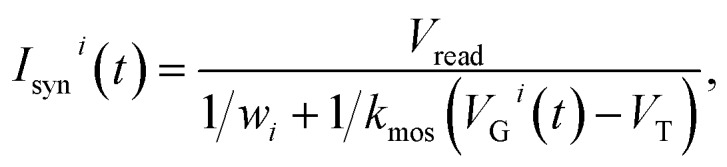
where *V*_read_ is the read voltage applied to the top electrode of the ReRAM, *w*_*i*_ is the weight (ReRAM conductance) of the *i*-th synapse, and *k*_mos_ and *V*_T_ are the parameters describing the transistor characteristics. The time-dependent total current entering the POST is determined by the temporal correlation of the two PRE spikes and the conductance of the two ReRAM devices. The CMOS POST circuit sums all the synaptic current by Kirchhoff’s law, and converts the total current into an internal potential 
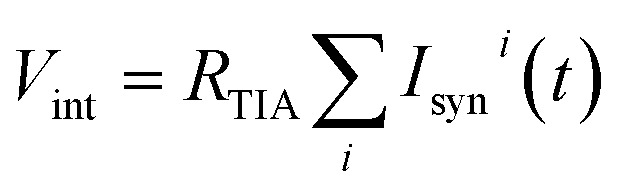
 by a trans-impedance amplifier (TIA) with a feedback resistance *R*_TIA_.

Fig. 2b shows the exponentially decaying signals of the output of the two PREs (upper panel) and the resulting *V*_int_ (lower panel), showing the incremental steps following each individual PRE spike, where each increment is proportional to the synaptic weight (the weights of the 2 ReRAM synapses for the demonstration are given in the inset of Fig. 2a). The voltage decay between each increment contributes to the overall evolution of the *V*_int_, which is responsible for the temporal correlation among the PRE spikes. Fig. 2c gives the maximum *V*_int_ as a function of *t*_c_. Note that similar results of the temporal computation can be obtained with regular spikes applied to the gate terminal of the synapse and with a leaky integrate & fire (LIF) POST neuron. Here we move the leakage function (decaying signal with time) to the PRE to mimic the shape of the action potential, as well as to enable the weight update algorithm related to the temporal information among the PRE spikes, as described in the following section.

### 2.3 Learning of the temporal correlation

We adopt a Widrow–Hoff (WH) learning rule for the weight update of the ReRAM synapses in our SNN during the training process. In the WH rule, each synaptic weight is updated according to a weight change given by,^39^

3 Δ*w*_*i*_ = *ηx*_i_(*y*_d_ – *y*_o_),

where *η* is the learning rate, *x*_i_ is the input variable, *y*_o_ is the output, and *y*_d_ is the expected output. Note that the output difference (*y*_d_ – *y*_o_) can be viewed as the error, which generally drives the supervised training of a multi-layer perceptron by gradient descent techniques.^15^ In the original WH rule, the variables (*x*_i_, *y*_d_ and *y*_o_) in eqn (3) are real-valued vectors. In SNN, the input and output signals are described by the spike timing, thus a WH-like learning rule for the precise-timing learning algorithm can be obtained by modifying eqn (3) as,^33^,^40^

4 Δ*w*_*i*_(*t*) = *ηV*_G_^*i*^(*t*)[*s*_d_(*t*) – *s*_o_(*t*)],

where *s*_d_(*t*) = *δ*(*t* – *t*_d_) and *s*_o_(*t*) = *δ*(*t* – *t*_o_) are the teacher signals of the supervisor circuit and the actual output spike of the POST, with *t*_d_ and *t*_o_ denoting the timing of the teacher spike and the actual output spike, respectively, and *δ*(*) being the Dirac delta function. The value of *s*_d_(*t*) – *s*_o_(*t*) can only be 0, –1, or 1, denoting the true fire, false fire, or false silence situations of the POST, respectively, thus guiding the weight update.

**Fig. 3:**
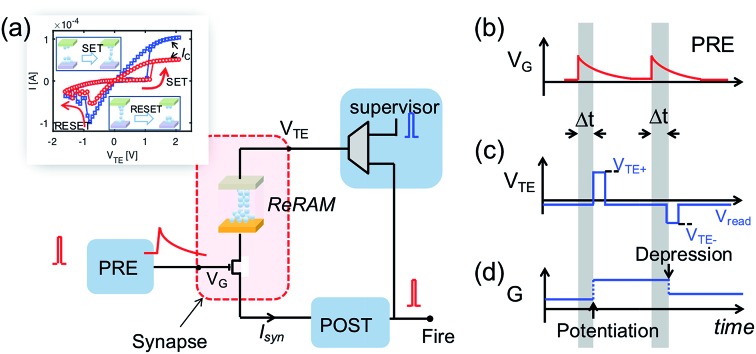
(a) The building block in a fully functional neuromorphic system implementing the weight update algorithm. Inset: The *I*–*V* characteristics of a single ReRAM device. (b–d) The weight updating rule implemented in the neuromorphic building block, showing: (b) the PRE signal applied to the gate terminal of 1T1R synapse; (c) the voltage applied to the top electrode of the ReRAM device, generated by the supervisor circuit; and (d) the weight update of the synapse. The direction of the weight update is decided by the polarity of the top electrode voltage, and the amount of weight change is related to the gate voltage of the transistor at the time of updating, which incorporates the temporal information of the PRE spikes.

Fig. 3 shows the implementation of the temporal weight update algorithm in eqn (4). The inset in Fig. 3a gives the two typical *I*–*V* curves of the ReRAM device, demonstrating the synaptic plasticity by the set/reset processes. With a high positive voltage applied on the top electrode (*V*_TE_) of the ReRAM device, the device can switch from a high resistance state (HRS) to low resistance state (LRS), also called set transition. On the other hand, when a high negative voltage is applied, the device can switch from LRS to HRS, which is called reset transition. The LRS conductance after set transition can be regulated by a compliance current *I*_C_, which is controlled by the gate voltage applied to the series transistor. In the reset transition, varying the gate voltage also controls the HRS conductance (not shown here), since a lower gate voltage results in a higher voltage drop between the source-drain terminals of the transistor, thus lowering the voltage drop across the ReRAM device.

Fig. 3b–d illustrate the operation scheme of the potentiation/depression of the synaptic weight in the building block. When the internal voltage *V*_int_ of POST exceeds a threshold *V*_th_, a spike is immediately generated and read by a supervisor circuit. The supervisor circuit compares the POST output signal with a teacher signal, which marks the presence of a ‘true’ sequence. There are three possible cases: (i) if the teacher signal and POST spike occur at the same time, this corresponds to a “true fire”, thus no weight update is needed; (ii) if the teacher signal occurs with no POST spike, this corresponds to a “false silence” case; (iii) if the POST spike occurs with no teacher signal, this corresponds to a “false fire” case. In the case of true fire (i), the top electrode voltage *V*_TE_ remains at the low level read voltage *V*_read_ (Fig. 3c). On the other hand, for the false silence (ii), a high positive voltage *V*_TE+_ is applied by the supervisor circuit to induce synaptic potentiation. Conversely, a high negative voltage *V*_TE–_ is applied to induce synaptic depression for the false fire case (iii). False fire/silence cases are shown in Fig. 3d, indicating that, in the correspondence of the *V*_TE+_ or *V*_TE–_ pulses, the exponentially decaying voltage on the transistor gate provides temporal information about the PRE spikes. As a result, the synaptic weight change is a function of the temporal information, allowing spatiotemporal learning.

### 2.4 Learning and recognition of the spike sequence

**Fig. 4:**
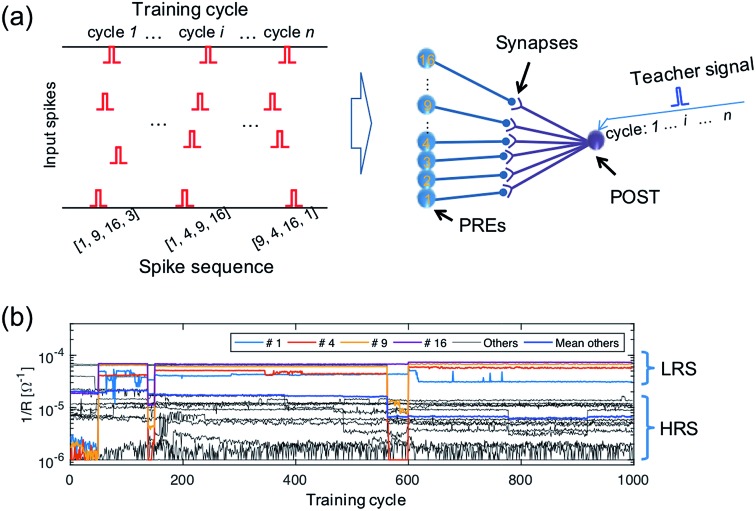
(a) A schematic illustration of the input spiking patterns submitted to a 16 × 1 spatiotemporal network supervised by a teacher signal. (b) The experimentally measured evolution of the synaptic weights during training.

To demonstrate learning and recognition of the spatiotemporal patterns, we considered spike sequences where neurons generate spikes sequentially with a fixed time interval. For instance, Fig. 4a shows the 4-spike sequences generated by 16 PREs, where the sequence [1, 4, 9, 16], namely the sequential spiking of the 1^st^, 4^th^, 9^th^, and 16^th^ PREs (cycle *i*), is considered as the ‘true’ sequence. Sequence learning was demonstrated by using a 16 × 1 spatiotemporal neuromorphic network, consisting of 16 PREs, 1 POST, and 16 ReRAM synapses. The goal of the training is that the POST spikes only in response to the true sequence [1, 4, 9, 16], while keeping silent in response to other sequences. During training, 4-spike sequences are submitted at each training cycle (Fig. 4a left panel), while the teacher signal generates a spike in correspondence of the true sequence [1, 4, 9, 16]. The input spikes and the teacher signals are provided by a microcontroller, although all the learning functions took place locally and independently at the CMOS-neuron/ReRAM synapse network. Fig. 4b shows the measured evolution of the synaptic weights during training: after training, the 1^st^, 4^th^, 9^th^, and 16^th^ synapses were potentiated to LRS, while other synapses were depressed to HRS. The four synapses in LRS show distinct conductance levels following the rule *w*_16_ > *w*_9_ > *w*_4_ > *w*_1_, which evidences the learning of the temporal information in the true sequence [1, 4, 9, 16].

**Fig. 5:**
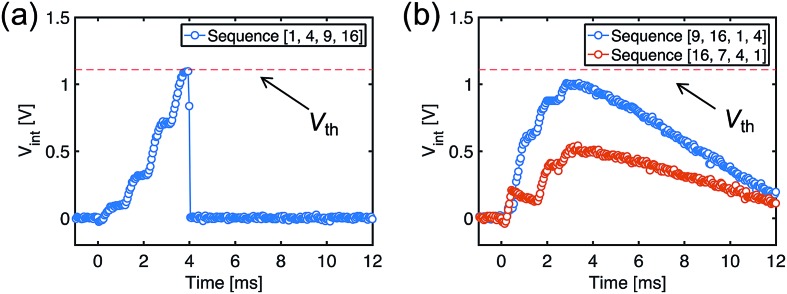
Measured *V*_int_, indicating spike accumulation by the POST for (a) the true sequence and (b) false sequences.

After the training process, we measured *V*_int_ in the POST in response to the submission of all the spike sequences (Fig. 5). For the true sequence, the successive accumulation of *V*_int_ reaches the threshold *V*_th_, thus leading to POST fire (Fig. 5a). The increments of *V*_int_ in correspondence with each PRE spike can be clearly seen, each step increase being determined by the corresponding ReRAM synapse weight. On the other hand, *V*_int_ remains below *V*_th_ for false sequences. For instance, *V*_int_ of the false sequence [16, 7, 4, 1] is far below the threshold (Fig. 5b), as a result of the 7^th^ synapse being in HRS. The permutations of the spiking pattern, *e.g.*, [9, 16, 1, 4], also lead to insufficient accumulation due to the time/weight mismatch.

### 2.5 Recognition of long sequences

The recognition of spiking sequences requires that the ReRAM synapses are potentiated to distinct LRS levels, thus longer spiking sequence might face the issue of the limited number of LRS levels in the ReRAM.^41^,^42^

**Fig. 6:**
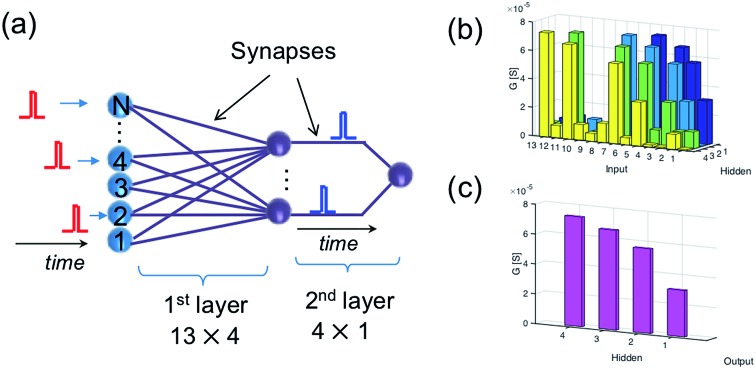
(a) Illustration of a two-layer spatiotemporal network to recognize a relatively long spiking sequence. (b and c) The conductance map of the synapses in the two-layer network.

A feasible solution to this issue is to introduce a multilayer neural network. For instance, Fig. 6a shows a neural network with 13 input neurons, 4 hidden neurons, and 1 output neuron, with 13 × 4 ReRAM synapses connecting the input neurons and hidden neurons in the first layer and 4 × 1 ReRAM synapses connecting the hidden neurons and output neuron. Fig. 6b and c shows the conductance map of the ReRAM synapses of the two-layer network, where the conductance states are assigned according to the result of the trained network in Fig. 4b. The network is designed to recognize the true spiking sequence of prime numbers from 1 to 13, *i.e.*, [1, 2, 3, 5, 7, 11, 13]. To this purpose, the first neuron in the hidden layer is designed to recognize the subsequence [1, 2, 3, 5], while the second neuron recognizes the subsequence [2, 3, 5, 7], and so on. The output neuron recognizes the successive spikes of the hidden neurons, thus leading to recognition of the long sequence [1, 2, 3, 5, 7, 11, 13].

**Fig. 7:**
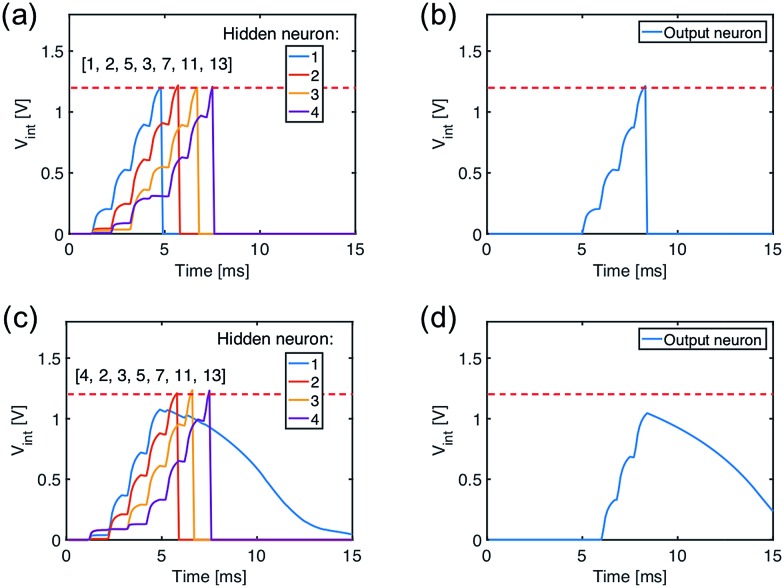
(a and b) The internal potential of the hidden layer neurons (a) and of the output neuron (b) under the submission of the true sequence. (c and d) The same, but under the submission of a false sequence.

Fig. 7a shows the internal potential *V*_int_ of the hidden layer neurons for the true sequence input, while Fig. 7b shows the response of the output neuron, where *V*_int_ reaches the threshold thus demonstrating sequence recognition. On the other hand, submission of a false sequence [4, 2, 3, 5, 7, 11, 13] does not reach the threshold in Fig. 7c and d, as a result of substituting the first ‘1’ with a ‘4’ causing the silence of the first neuron in the hidden layer.

Note that the maximum value of *V*_int_ (Fig. 5b and 7d) indicate the similarities of the test sequences with the true sequence, enabling even the toy neural network with wide application potential. For instance, with 26 PREs representing one letter each, the network can be used to check spelling errors of word. The *V*_int_ similarities of sequences can be compared with the Damerau–Levenshtein (DL) distance^43^,^44^ of sequences, which is widely used in spell checking, speech recognition, DNA analysis, *etc.* The calculation of the DL distance requires many steps of comparing each element of the sequences within at least two programming loops.^43^ On the other hand, the POST *V*_int_ can assess the similarity between the patterns with analog behavior, and only requires one inference step in a spatiotemporal SNN after training.

### 2.6 Learning of a spiking sequence

**Fig. 8:**
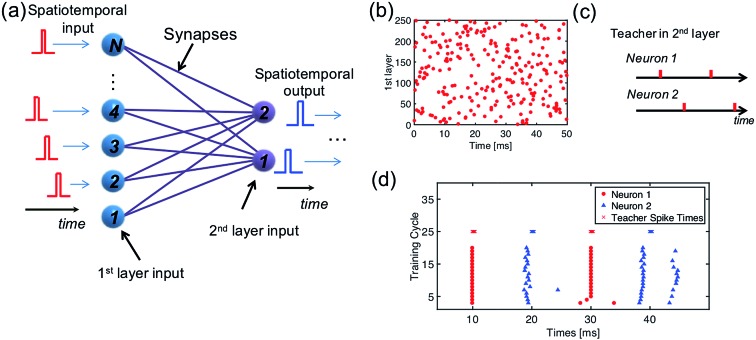
(a) Illustration of a neural network for the mapping of a spatiotemporal pattern. (b) Randomly generated spatiotemporal input pattern; (c) spatiotemporal output pattern; (d) the training of mapping a complex spatiotemporal pattern to a simple one.

In a multilayer spatiotemporal network, the output spikes of one layer must be spatiotemporally-coded to act as the input of the next layer.^45^ Though a complete training algorithm of a multilayer spatiotemporal network is still missing, training the network to map a spatiotemporal input into a spatiotemporal output (Fig. 8a) is a critical step.^46^Fig. 8b shows a randomly generated spatiotemporal input pattern for 250 input neurons, and Fig. 8c shows a spatiotemporal pattern as the training target of the two output neurons. To associate these two spatiotemporal spiking patterns, a network consisting of 250 PREs, 2 POSTs, and 250 ReRAM synapses is needed. Following the same learning rule shown in Section 2.3, the 250 × 2 neural network is successfully trained to generate the target spiking pattern when the input spatiotemporal pattern was presented (Fig. 8d).

### 2.7 Detection of a moving object

**Fig. 9:**
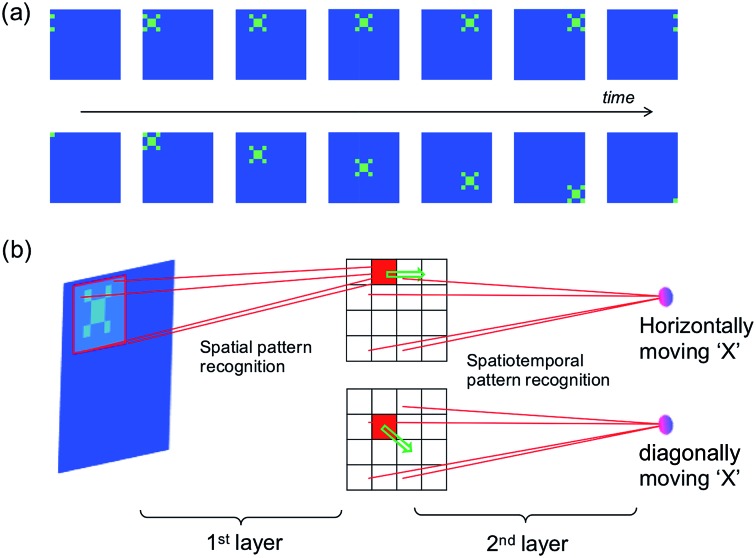
(a) Illustration of a dynamic “X” pattern moving horizontally (first row) or diagonally (second row). (b) Illustration of the two-layer neural network for the recognition of a moving object.

The spatiotemporal sequence learning and recognition lies at the basis of the ability to interact with a dynamic environment, *e.g.*, for speech and gesture recognition. Fig. 9a illustrates the detection of moving objects by a spatiotemporal network, where the moving trace of pattern “X” can be represented by a spatiotemporal spiking pattern with each spiking neuron corresponding to the position of the pattern “X” at any given time. Fig. 9b shows the neural network for movement detection, where the first layer is a conventional spatial-pattern network for the recognition of the pattern ‘X’,^8^ while the second layer is a spatiotemporal network to detect the trace of the pattern.

The spatial network can be trained, for instance, by unsupervised learning method,^8^ so that only the pattern “X” would induce a spike in the corresponding inter-layer neuron representing the position of “X”. The moving object, then, would result in a sequential spiking of the interlayer neurons. Output neurons can thus be trained to recognize spatiotemporal sequences representing the various directions of the pattern.

### 2.8 Spatiotemporal network for pattern recognition

**Fig. 10:**
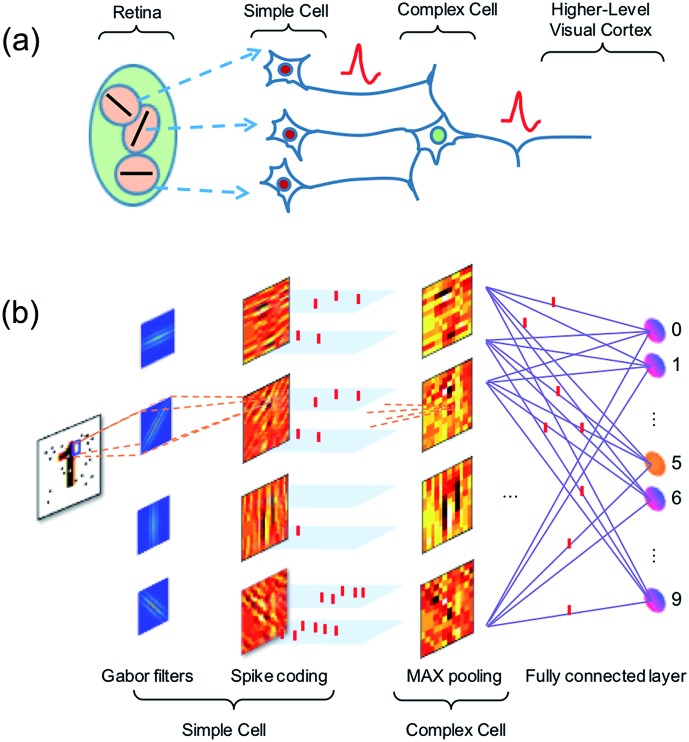
(a) Illustration of the hierarchy structure of the biological visual system. (b) Schematic diagram of the artificial visual system for feature extraction and classification using spatiotemporal spike coding.

Vision in the mammalian brain follows hierarchical rules,^47^ where signals from the retina are first projected to simple cells with orientation sensitivity to extract the basic features of the image, then more complex cells are used to increase the visual system’s invariant to the input image. Finally, the higher-level cortex participates in the detection of basic features in the image, enabling pattern recognition (Fig. 10a).^48^

Here, we propose an artificial visual system (Fig. 10b), where Gabor filters with various sizes and orientations are used to mimic the receptive fields of the simple cells. The output of the simple cells is converted into spatiotemporal spikes by amplitude–time conversion, *i.e.*, the neuron with the highest signal spikes first. Then max-pooling neurons acting as complex cells select the most salient features in nearby receptive fields. The spatiotemporal patterns from complex cells are finally used to train a fully-connected spatiotemporal network.

**Fig. 11:**
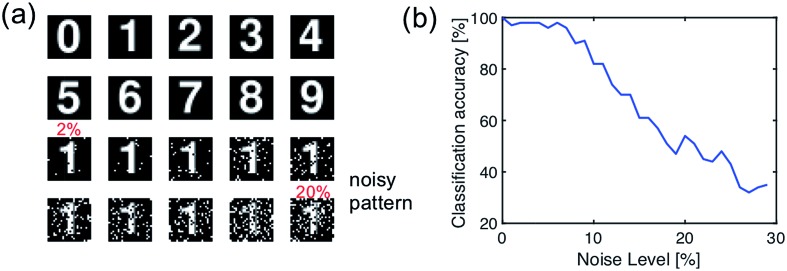
(a) The optical digital character for training (clean pattern) and testing (noise pattern) of the artificial hierarchical visual neural network. (b) The recognition rate of the trained network as a function of the noise level of the optical digital character.

To validate this artificial visual system, we used a simple pattern recognition task, *i.e.*, optical character recognition (OCR). The synapses of the fully connected layer are initially prepared in a random conductance map. The network is trained with the ideal character image (Fig. 11a), then inference was tested with a set of noisy patterns. Fig. 11b shows the results of the testing, in terms of the recognition rate of noise pattern as a function of the noise level. The recognition rate remains higher than 90% percentage with the noise level lower than 7%, thus demonstrating the accuracy of spatiotemporal coding for spatial pattern recognition.

## 3 Experimental

### 3.1 ReRAM synapse

The ReRAM device^49^ used in this study consists of a 10 nm thick switching layer of Si-doped HfO_*x*_ deposited by atomic layer deposition (ALD) on a confined 50 nm diameter TiN bottom electrode and a Ti top electrode on the HfO_*x*_ dielectric layer. A forming operation was initially conducted by the application of a pulse of 3 V amplitude and 100 ms pulse width to initiate the conductive filament path. The ReRAM was connected *via* the bottom TiN electrode to a field-effect-transistor, which was integrated in the front-end of the same silicon chip by a conventional CMOS process. The resulting 1T1R structure was controlled during forming, set, and reset processes by applying pulses to the top electrode and gate contacts, with grounded source contact.

### 3.2 CMOS neuron

In the network, each PRE represents a neuron cell and its axon terminal. Each axon terminal is connected to the gate terminal of a 1T1R synapse. All synaptic top electrodes were controlled by CMOS circuits providing a constant bias *V*_read_ = –0.3 V to induce the synaptic current *I*_syn_ in response to an axon spike.^8^ All source terminals were connected to the POST input (virtually grounded),^14^,^25^,^50^ consisting of a trans-impedance amplifier (TIA) to convert the summed synaptic currents ∑*I*_syn_ into the internal potential *V*_int_. The latter was compared with the threshold voltage *V*_th_ to induce fire for *V*_int_ > *V*_th_. During supervised training, a teacher spike was applied to the top electrode to induce potentiation or depression. The voltage of the top electrodes was switched from *V*_read_ to a pulse of positive voltage *V*_TE+_ = 3 V after false silence or negative voltage *V*_TE–_ = –1.6 V after false fire to induce time-dependent potentiation or depression, respectively.

### 3.3 Training and test control system

The synaptic network was connected to an Arduino Due microcontroller (μC) on a PCB for the training and testing of the synaptic network. To operate the network, the PRE spike sequence was first stored in the internal memory of the μC, then the sequence was launched while monitoring the synaptic weights and internal potential *V*_int_ at each cycle. The spike and fire potential and input currents were also monitored by a Lecroy Waverunner oscilloscope. The μC only provides spiking information (including the teacher signal) during the training and test stage, whereas all learning processes, *i.e.*, the adjustment of synaptic, were achieved by the network of hybrid CMOS-neurons/ReRAM synapses in real time. For best accuracy in our PCB system, we adopted an axon potential decay constant *τ* = 8 ms.

## 4 Conclusions

ReRAM devices are among the most promising technologies for artificial synapses in neuromorphic computing systems. To construct an artificial neural network competing with the brain’s functionality and efficiency, a ReRAM based SNN that can replicate the temporal computing in the brain is critical. This work addresses the methodology and hardware implementation of a neuromorphic SNN system to compute the temporal information among neural spikes using ReRAM synapses capable of STDP. We first experimentally demonstrate the learning and recognition of spatiotemporal-coded spike sequences, enabling the wide application potential, *e.g.*, spell checking and DNA analysis. Cascade spatiotemporal computing within the multilayer networks of ReRAM synapses are also presented. Utilizing the temporal computing between spikes, it is possible to learn and detect the trace of a moving object. We then design and simulate a pattern recognition system mimicking the hierarchical structure of the biological visual cortex using ReRAM synapses and the proposed methodology. The results confirm the ability of the temporal computing of ReRAM synapses and the feasibility of ReRAM synapses for the hardware implementation of “brain-like” neuromorphic system with efficient spatiotemporal coding.

## Conflicts of interest

There are no conflicts to declare.
